# Supplementary material for: BAD regulates mammary gland morphogenesis by 4E-BP1-mediated control of localized translation in mouse and human models
Source: Nat Commun. 2021 May 19;12:2939. doi: 10.1038/s41467-021-23269-8 (PMC8134504; doi:10.1038/s41467-021-23269-8)
Supplement: Supplementary file 1 — Supplementary Information [file 41467_2021_23269_MOESM1_ESM.pdf]

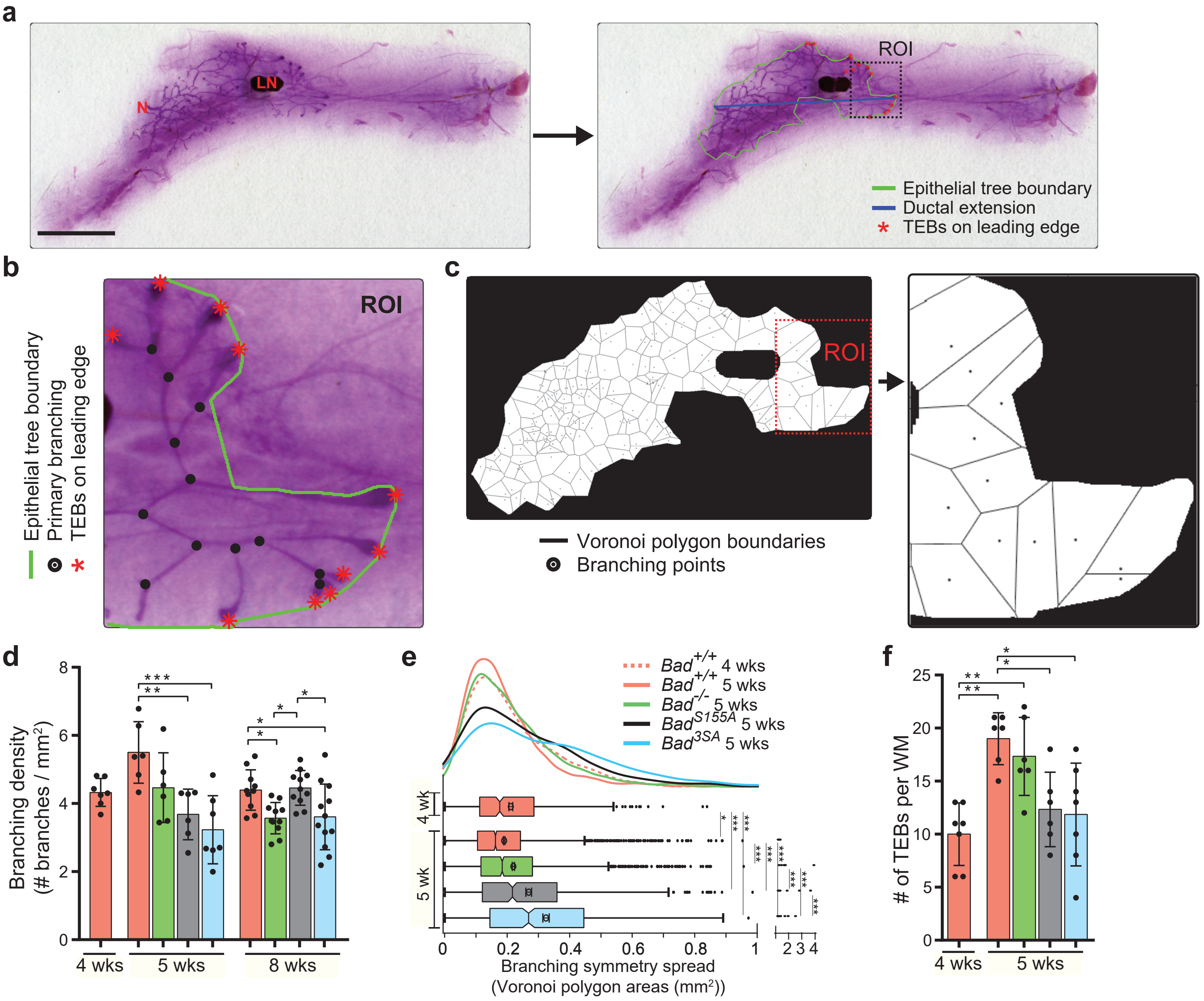

Supplementary Figure 1. Whole mount analysis of all genotypes

a) Sample features detected from a digitized WM image. Green boundary shows area occupied by the epithelial tree. Blue line shows the longest distance between N (tree origin) and tree boundary, indicative of ductal extension. Red asterisks show terminal end buds (TEBs) on the leading edge. N, nipple; LN, lymph node; Scale bars = 5mm. **b)** Magnification of the region of interest (ROI) from a, highlighting epithelial tree boundary, primary branching points and TEBs on the leading edge. **c)** Typical Voronoi tessellation diagram computed from the primary branch points to examine overall ductal organization. **d-f)** Additional morphological feature analysis of WMs from Fig. 2 b-c. For e, Tukey boxplots highlights distribution of branching points Voronoi polygons areas from the WMs. The areas would have a narrow range spread for symmetrical branching patterns. *Bad*^{3SA} glands showed the widest range of Voronoi areas followed by *Bad*^{S155A}, indicating that loss of phosphorylation produced an inconsistent branched network. On top of the boxplots is Kernel fitted normalized histograms of the Voronoi areas distribution. For Tukey boxplots, constriction indicates median, notch indicates 95% confidence interval, box edges are 25th and 75th percentiles, whiskers show extreme data points, 'outliers' plotted as black dots, overlaid circle and error bars are the mean ± SEM. For all *p*-values, *** P<0.001, ** P<0.01, * P<0.05. Statistical test details and exact *p*-values are provided in Sup. Data 4. Source data are provided in the Source Data file.

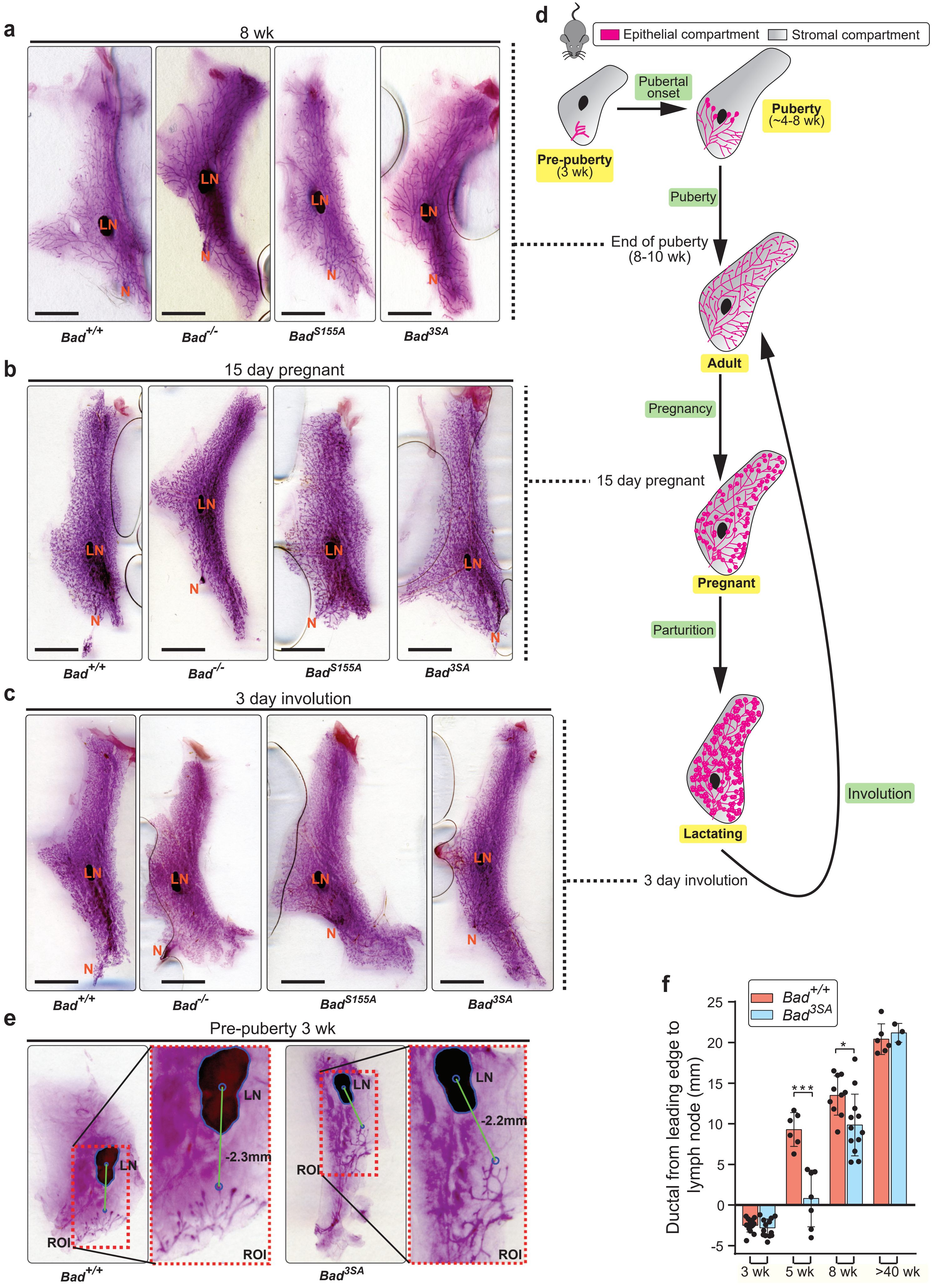

a
8 wk
LN
N
Bad+/+
Bad-/-
BadS155A
Bad3SA
b
15 day pregnant
c
3 day involution
d
Epithelial compartment
Stromal compartment
Pre-puberty (3 wk)
Pubertal onset
Puberty (~4-8 wk)
Puberty
End of puberty (8-10 wk)
Adult
Pregnancy
15 day pregnant
Pregnant
Parturition
Lactating
Involution
3 day involution
e
Pre-puberty 3 wk
ROI
-2.3mm
-2.2mm
f
Ductal from leading edge to lymph node (mm)
Bad+/+
Bad3SA
***
*
3 wk
5 wk
8 wk
>40 wk

Supplementary Figure 2. Mammary gland whole mounts at different stages of mammary gland development of all genotypes

a-c) Representative carmine stained WMs of mammary gland #4 from 8wk mice (approaching end of puberty), 15-day pregnant mice (pregnancy stage) and 3-day involution post-weaning (involution stage), respectively. Minimum of 3 independent mice were assessed per developmental stage. Scale bars = 5mm. **d)** Schematic for the different developmental stages of the mouse mammary gland, with the dotted lines highlighting corresponding representative WMs. **e)** Representative carmine stained WMs of excised pre-pubertal mammary gland #4 from 3 wk mice (see schematic in Fig.2d. $Bad^{+/+}$ *n*=14, Bad^{3SA} *n*=13). Blue boundary highlights LN while green line shows the shortest distance between LN centroid and epithelial leading edge, indicative of ductal extension. **f)** Bar plots of distance from LN centroid to the epithelial leading-edge (mean ± s.d.) of $Bad^{+/+}$ and Bad^{3SA} WMs from different developmental stages. For 3 wk, 5 wk, 8 wk and old virgins (>40 wk), $Bad^{+/+}$ *n*=14, 6, 10, 6 and Bad^{3SA} *n*=13, 7, 12, 3 independent mice respectively. For all *p*-values, *** P<0.001, ** P<0.01, * P<0.05. Statistical test details and exact *p*-values are provided in Sup. Data 4. Source data are provided in the Source Data file.

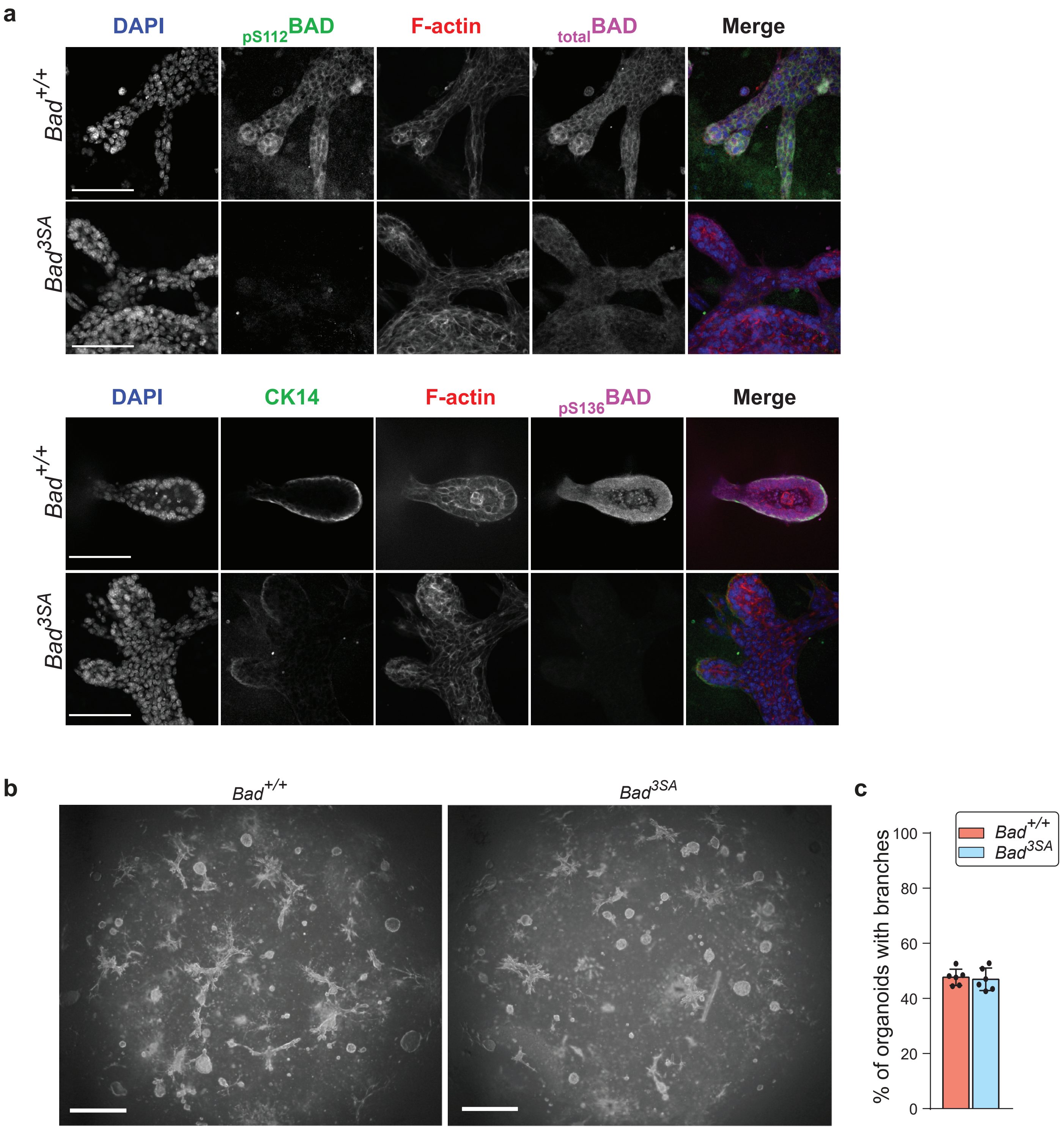

Supplementary Figure 3. Mouse 3D branching organoid immunofluorescence and bright field analyses

a) Representative immunofluorescence images from 3 independent mice, of mouse branching organoids (day 5) stained for total or pS136-BAD (magenta), pS112-BAD or CK14 (green), F-actin (red) and DAPI (blue). Scale bars = 100µm. **b)** Representative low magnification phase contrast imaging of mouse branching organoids embedded in Matrigel:Collagen-I gels from 3 independent experiments. *Bad*$^{+/+}$ *n*=6, *Bad*^{3SA} *n*=6 fields. Scale bars = 1mm. **c)** Bar plots for percentage of organoids branching, from 3 independent experiments. *Bad*$^{+/+}$ *n*=6, *Bad*^{3SA} *n*=6 fields. Source data are provided in the Source Data file.

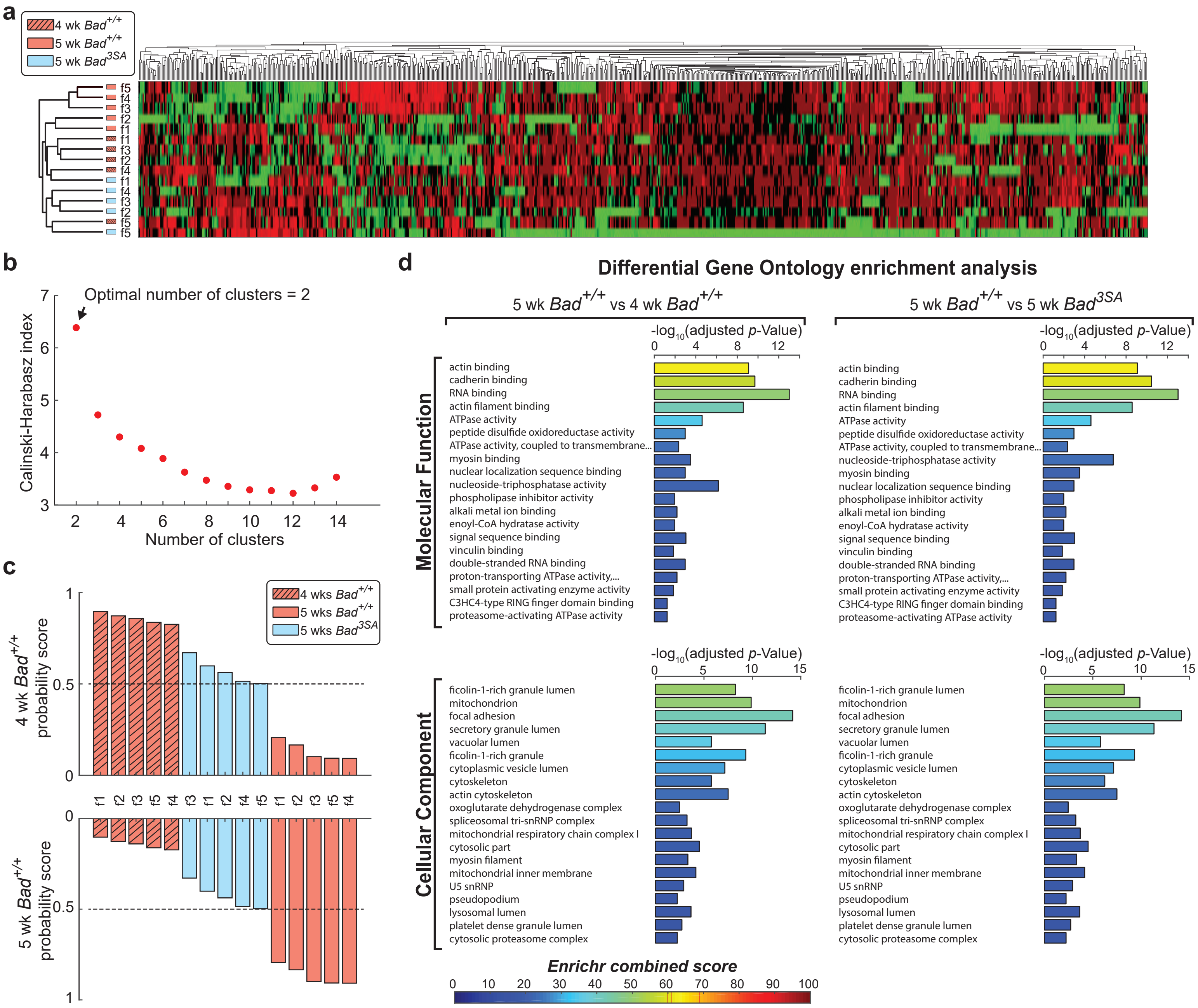

Supplementary Figure 4. MS proteomic screen analysis

a) Unsupervised hierarchical correlation clustering of Mass Spectrometry protein profiles from pubertal onset Bad$^{+/+}$ (4 wk) and puberty *Bad$^{+/+}$* or *Bad^{3SA}* (5 wk) lysates. *n*=799 proteins (See 'Methods'). **b)** Optimal number of clusters from the protein profile data, determined using Calinski-Harabasz criterion. **c)** Probability scores (probability of the profile originating from the training data class) for all sample's protein profile data, determined by machine learning random forest classification (*Bad$^{+/+}$* 4 wk vs *Bad$^{+/+}$* 5 wk trained classification, 90% prediction accuracy. See 'Methods'). **d)** Gene Ontology analysis of differential proteins between *Bad$^{+/+}$* 5 wk and *Bad$^{+/+}$* 4 wk or *Bad^{3SA}* 5 wk. Enricher combined scores (bar heatmap color) and adjusted *p*-values (See 'Methods') highlight significance.

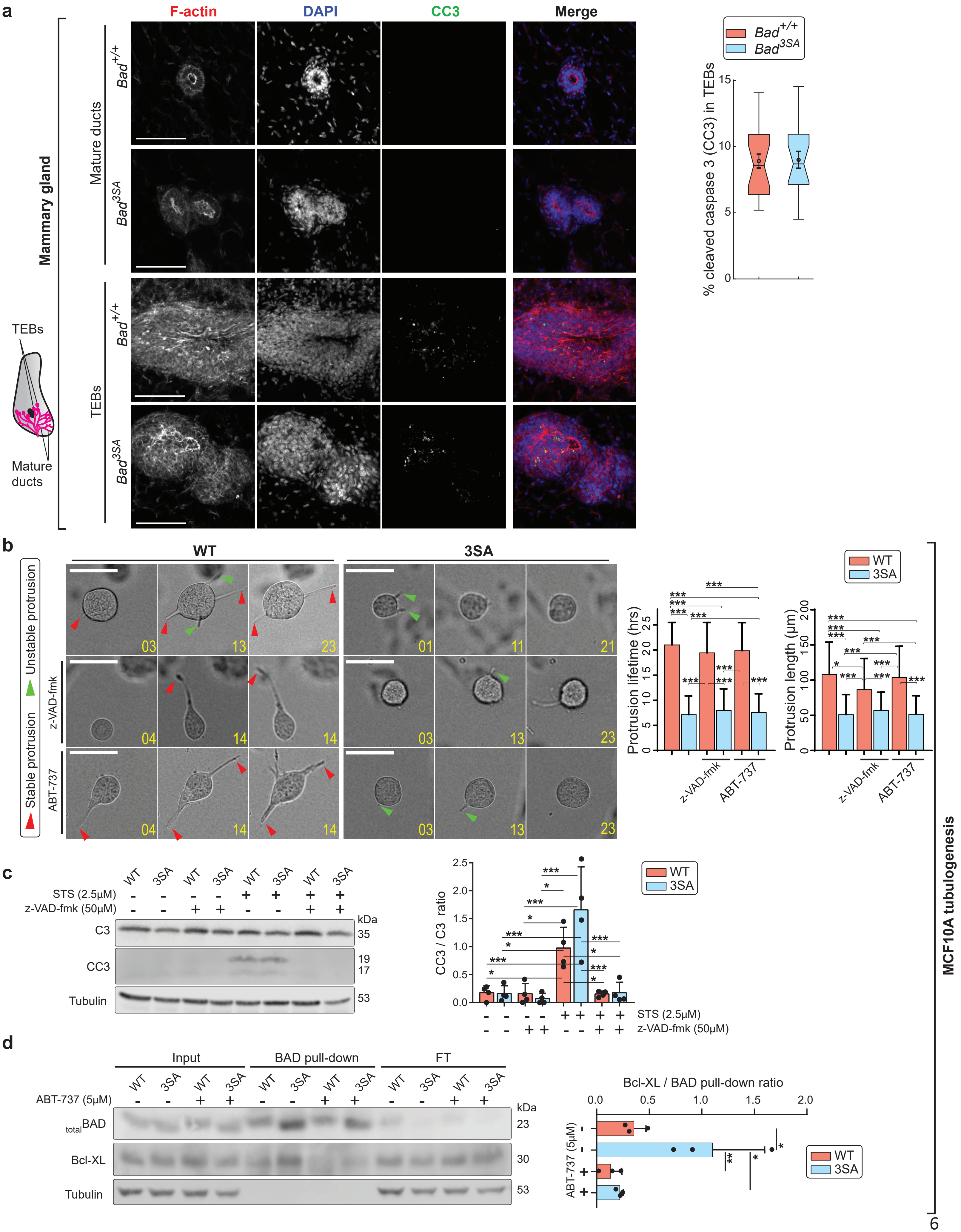
a
F-actin
DAPI
CC3
Merge
Mammary gland
Mature ducts
TEBs
Bad+/+
Bad3SA
Mature ducts
% cleaved caspase 3 (CC3) in TEBs
b
WT
3SA
Unstable protrusion
Stable protrusion
z-VAD-fmk
ABT-737
Protrusion lifetime (hrs)
Protrusion length (µm)
c
STS (2.5µM)
z-VAD-fmk (50µM)
C3
CC3
Tubulin
kDa
CC3 / C3 ratio
d
Input
BAD pull-down
FT
ABT-737 (5µM)
totalBAD
Bcl-XL
Tubulin
Bcl-XL / BAD pull-down ratio
MCF10A tubulogenesis

Supplementary Figure 5. BAD 3SA-defective tubulogenesis is caspase-independent and does not require Bcl-XL interaction

a) Left: Representative immunofluorescence images of pubertal (5 wk) mammary gland cryosections stained for cleaved caspase 3 (CC3). Regions representing mature ducts (upper) or TEBs (lower) within the same tissue sections are shown. F-actin (red), CC3 (green) and DAPI (blue). Scale bars = 100µm. Right: Tukey boxplot for percentage of cells positive for CC3 in TEBs showed no difference between the genotypes. *Bad*$^{+/+}$ *n*=22 TEBs, *Bad*^{3SA} *n*=18 TEBs from 3 independent mice per genotype. **b)** Left: Representative bright-field time-lapse images for 3D tubulogenesis assays of WT and 3SA cells, with or without z-VAD-fmk or ABT-737. Imaging and drug incubation were started on day 3 of tubulogenesis assays. Yellow numbers in insets indicate timepoint (hrs) during imaging (For complete time-lapse images, see Sup. Mov. 2). Red arrowheads indicate stable protrusions and green arrowheads indicate unstable protrusions. Scale bars = 100µm. Right: Quantitation of average protrusion lifetime and average protrusion length shows that neither z-VAD-fmk nor ABT-737 treatment rescues protrusion stability. WT no treatment *n*=56, WT z-VAD-fmk *n*=92, WT ABT-737 *n*=51, 3SA no treatment *n*=58, 3SA z-VAD-fmk *n*=61, 3SA ABT-737 *n*=41, from 3 independent experiments. **c)** Left: Representative western blot of lysates from MCF10A 3D tubulogenesis assay (day 4) that had been pretreated with z-VAD-fmk for 24h or staurosporine (STS) for 4h. Blots were probed for full-length or cleaved caspase 3 (C3 and CC3, respectively). Right: Quantitation shows STS-induced caspase 3 cleavage is similar between the genotypes and is inhibited by zVAD-fmk. *n*=4 and data are mean $\pm$ SD. **d)** Left: Representative western blot of MCF10A tubulogenesis lysates (day 4) immunoprecipitated for BAD to assess association with Bcl-XL. Where indicated, cells were incubated with or without ABT-737 for 24 hours. Right: Quantitation of Bcl-XL/BAD ratio shows increased association of Bcl-XL with 3SA relative to WT, which is reduced by ABT-737. *n*=3. Data are mean $\pm$ SD. For Tukey boxplots, constriction indicates median, notch indicates 95% confidence interval, box edges are 25th and 75th percentiles, whiskers show extreme data points, 'outliers' plotted as black dots, overlaid circle and error bars are the mean ± SEM. For all *p*-values, *** P<0.001, ** P<0.01, * P<0.05. Statistical test details and exact *p*-values are provided in Sup. Data 4. Source data are provided in the Source Data file.

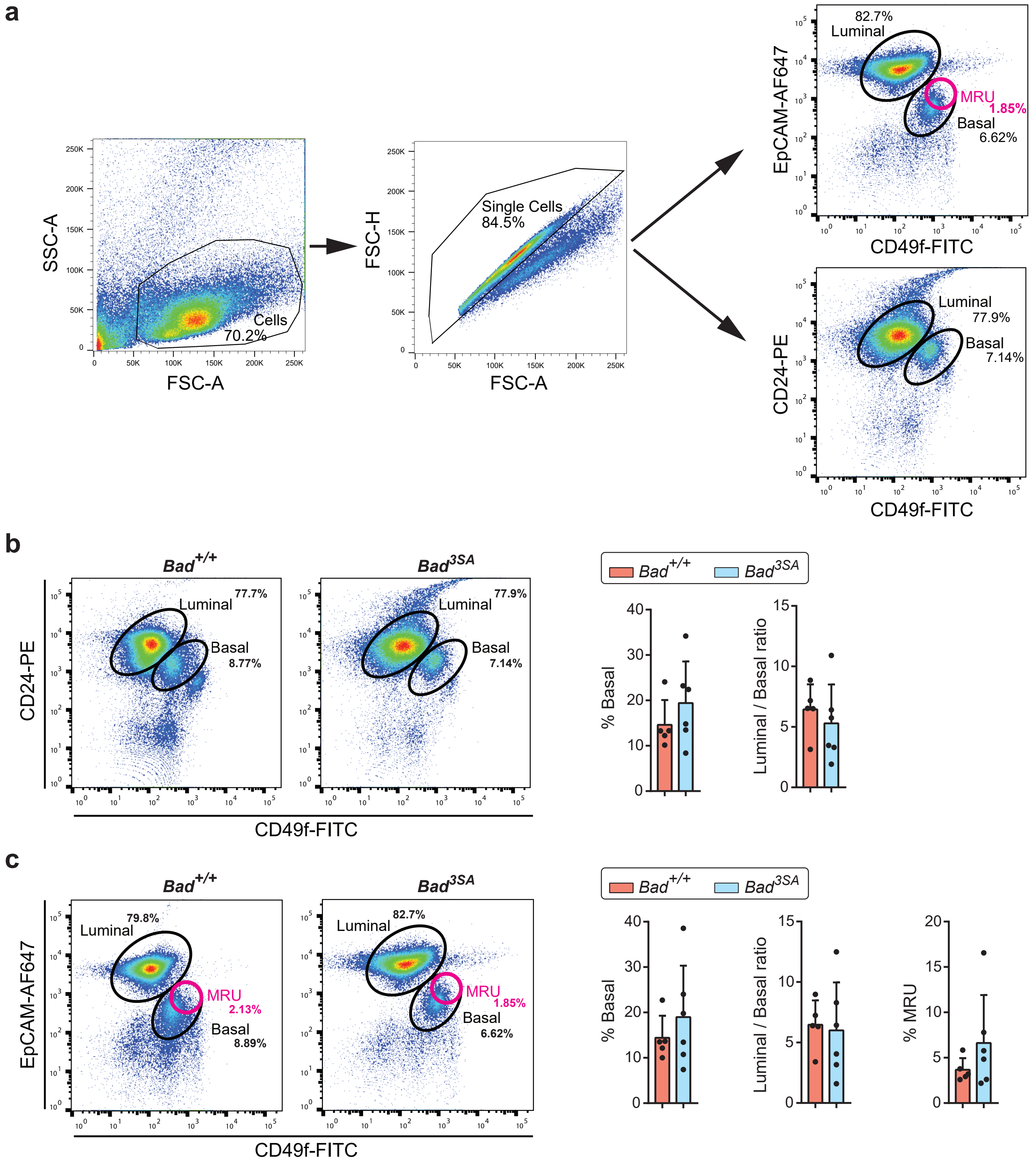

Supplementary Figure 6. Flow cytometry analysis and cell lineages determination

a) FACs pre-gating for single cells after excluding lineage negative cells (see 'Methods'). Mammary epithelial cell lineage was determined using EpCAM, CD49f and CD24 marker analysis. **b-c)** Left: Representative visualization of the luminal (EpCAM^{high}CD49f^{med} or CD24^{high}CD49f^{med}) and basal (EpCAM^{med}CD49f^{high} or CD24^{med}CD49f^{high}) cell populations. Magenta ROI highlights Mammary Repopulating Unit subpopulation (MRU). Right: The percentage of basal cells, ratio of luminal to basal cells and percentage of MRU are shown as mean ± SD. Number of animals: *Bad*$^{+/+}$ *n*=5, *Bad*^{3SA} *n*=6. For all *p*-values, *** P<0.001, ** P<0.01, * P<0.05. Statistical test details and exact *p*-values are provided in Sup. Data 4. Source data are provided in the Source Data file.

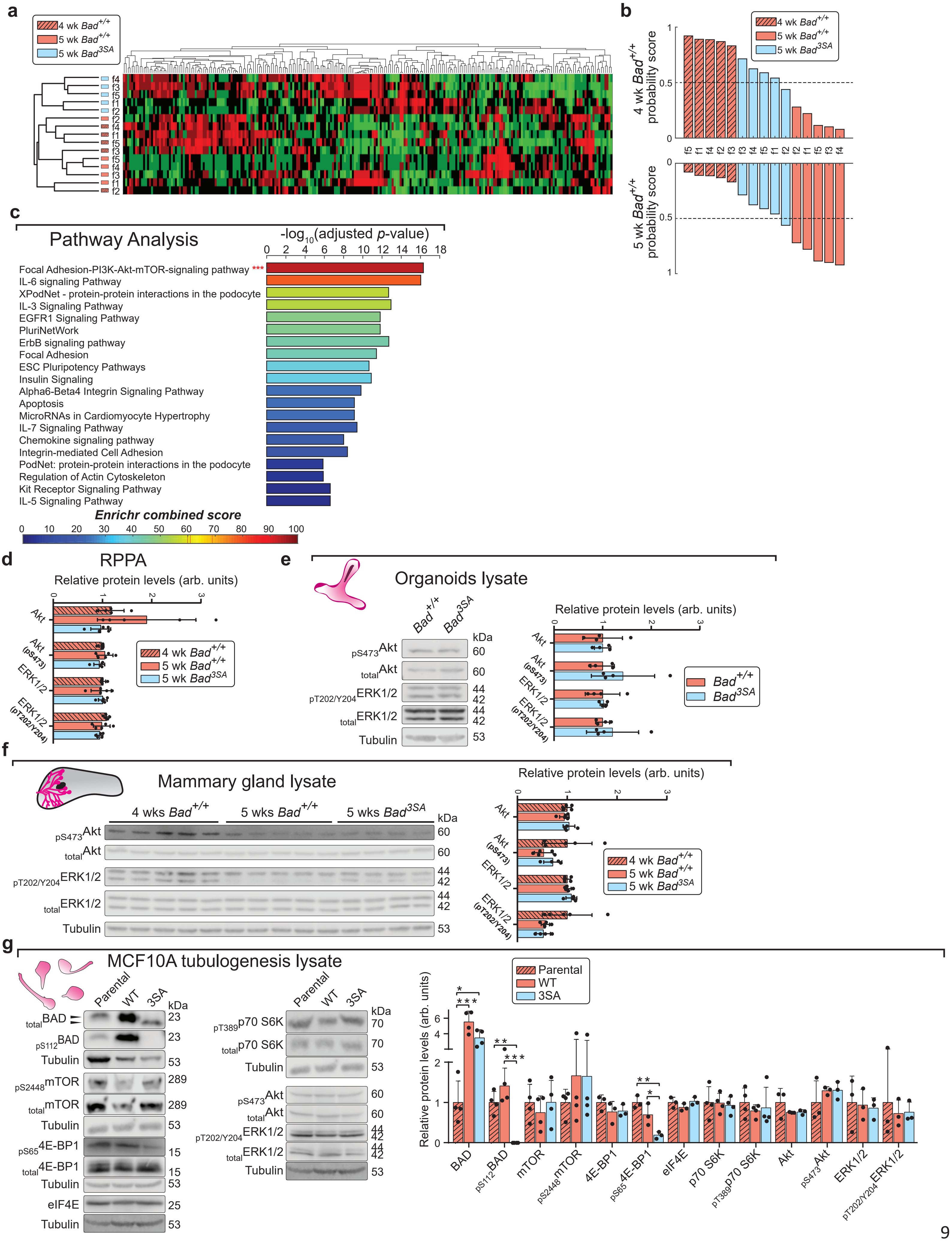
a
4 wk Bad+/+
5 wk Bad+/+
5 wk Bad3SA
f4
f3
f5
f1
f2
f2
f4
f1
f5
f3
f5
f4
f3
f1
f2
b
4 wk Bad+/+ probability score
5 wk Bad+/+ probability score
c
Pathway Analysis
-log10(adjusted p-value)
0 2 4 6 8 10 12 14 16 18
Focal Adhesion-PI3K-Akt-mTOR-signaling pathway ***
IL-6 signaling Pathway
XPodNet - protein-protein interactions in the podocyte
IL-3 Signaling Pathway
EGFR1 Signaling Pathway
PluriNetWork
ErbB signaling pathway
Focal Adhesion
ESC Pluripotency Pathways
Insulin Signaling
Alpha6-Beta4 Integrin Signaling Pathway
Apoptosis
MicroRNAs in Cardiomyocyte Hypertrophy
IL-7 Signaling Pathway
Chemokine signaling pathway
Integrin-mediated Cell Adhesion
PodNet: protein-protein interactions in the podocyte
Regulation of Actin Cytoskeleton
Kit Receptor Signaling Pathway
IL-5 Signaling Pathway
Enrichr combined score
0 10 20 30 40 50 60 70 80 90 100
d
RPPA
Relative protein levels (arb. units)
Akt
Akt (pS473)
ERK1/2
ERK1/2 (pT202/Y204)
e
Organoids lysate
Bad+/+
Bad3SA
kDa
pS473Akt 60
totalAkt 60
pT202/Y204ERK1/2 44 42
totalERK1/2 44 42
Tubulin 53
Relative protein levels (arb. units)
f
Mammary gland lysate
4 wks Bad+/+
5 wks Bad+/+
5 wks Bad3SA
kDa
pS473Akt 60
totalAkt 60
pT202/Y204ERK1/2 44 42
totalERK1/2 44 42
Tubulin 53
Relative protein levels (arb. units)
g
MCF10A tubulogenesis lysate
Parental
WT
3SA
kDa
totalBAD 23
pS112BAD 23
Tubulin 53
pS2448mTOR 289
totalmTOR 289
Tubulin 53
pS654E-BP1 15
total4E-BP1 15
Tubulin 53
eIF4E 25
Tubulin 53
pT389p70 S6K 70
totalp70 S6K 70
Tubulin 53
pS473Akt 60
totalAkt 60
pT202/Y204ERK1/2 44 42
totalERK1/2 44 42
Tubulin 53
Relative protein levels (arb. units)
BAD
pS112BAD
mTOR
pS2448mTOR
4E-BP1
pS654E-BP1
eIF4E
p70 S6K
pT389p70 S6K
Akt
pS473Akt
ERK1/2
pT202/Y204ERK1/2

Supplementary Figure 7. RPPA screen analysis and validation

a) Unsupervised hierarchical correlation clustering of RPPA protein/phosphoproteins profile from pubertal onset *Bad*$^{+/+}$ 4 wk and pubertal *Bad*$^{+/+}$ and *Bad*^{3SA} 5 wk lysates. *n*=246 protein/phosphoproteins (See Sup. Data 2 and 'Methods'). **b)** Probability scores for all samples' protein/phosphoproteins profile data, determined by machine learning random forest classification (*Bad*$^{+/+}$ 4 wk vs *Bad*$^{+/+}$ 5 wk trained classification, 90% prediction accuracy). **c)** Pathway analysis of the RPPA differential protein/phosphoproteins. Enricher combined scores (bar heatmap color) and adjusted p-value (See 'Methods') highlight significance. **d)** Total/phospho Akt, ERK1/2 levels from RPPA results. *Bad*$^{+/+}$ 4 wk *n*=5, *Bad*$^{+/+}$ 5 wk *n*=5, *Bad*^{3SA} 5 wk n=5 independent mice. Data are mean ± SD. **e)** Left: Western blot validation for total/phospho Akt, ERK1/2 levels. New independent mice were used for validation lysates (*Bad*$^{+/+}$ 4 wk *n*=5, *Bad*$^{+/+}$ 5 wk *n*=5, *Bad*^{3SA} 5 wk *n*=4). Right: Bar plots for the blots (mean ± SD). **f)** Left: Western blot validation for total/phospho Akt, ERK1/2 using lysates from mouse 3D branching organoid assay gels on day 6. Right: Bar plots for the blots from 4 independent experiments (mean ± SD). **g)** Left: Western blots of tubulogenesis lysates (day 7) for validation of proteins probed in Fig. 5d as well as total/phospho Akt, ERK1/2 levels. Black arrows highlight gel shift indicative of BAD phosphorylation. Right: Bar plots for the blots from 3 to 4 independent experiments (mean ± SD). For all *p*-values, *** P<0.001, ** P<0.01, * P<0.05. Statistical test details and exact *p*-values are provided in Sup. Data 4. Source data are provided in the Source Data file.

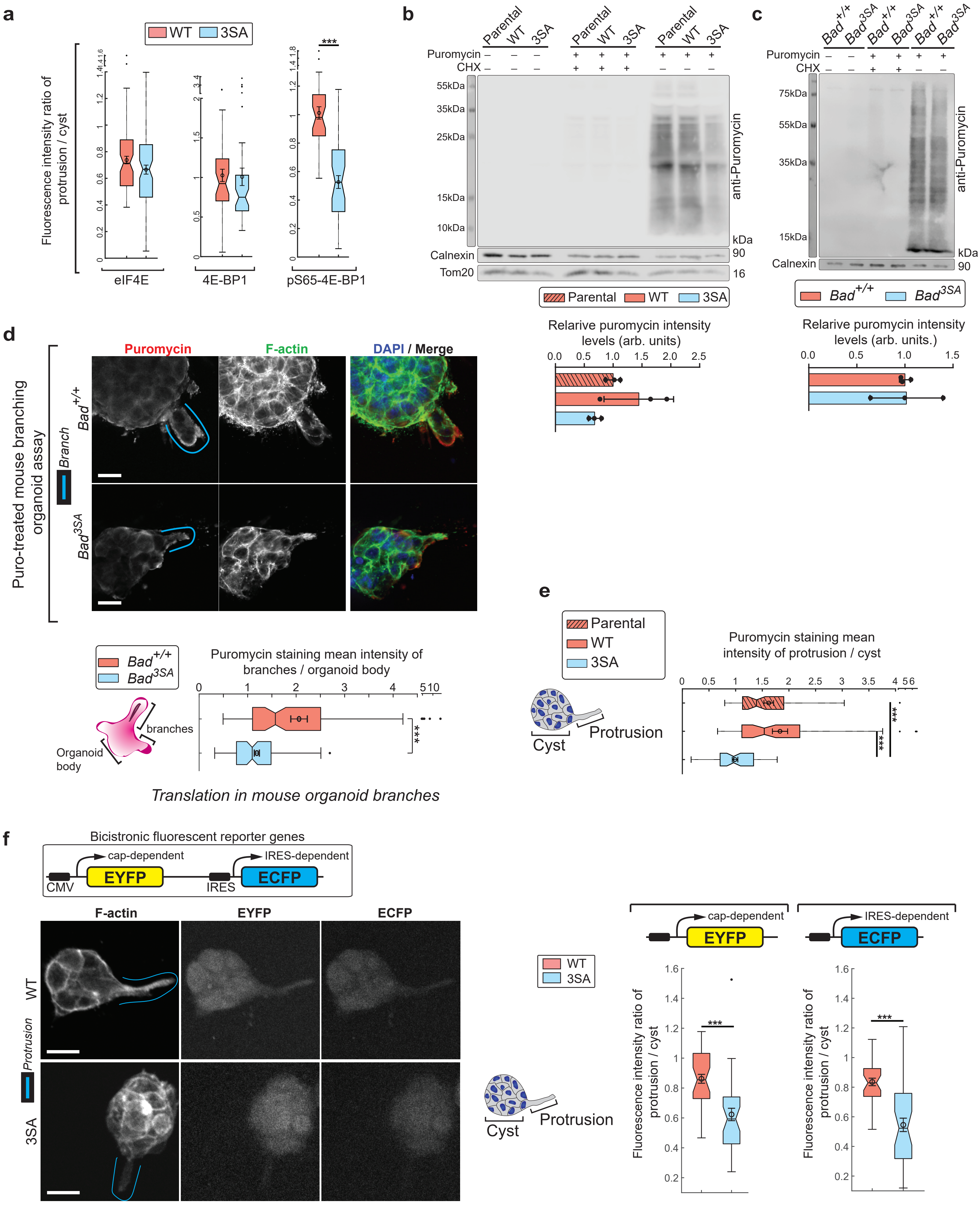

a
WT
3SA
Fluorescence intensity ratio of protrusion / cyst
eIF4E
4E-BP1
pS65-4E-BP1
***
b
Parental
WT
3SA
Puromycin
CHX
55kDa
35kDa
25kDa
15kDa
10kDa
kDa
anti-Puromycin
Calnexin
90
Tom20
16
Relarive puromycin intensity levels (arb. units)
c
Bad+/+
Bad3SA
75kDa
55kDa
35kDa
15kDa
Relarive puromycin intensity levels (arb. units.)
d
Puromycin
F-actin
DAPI / Merge
Puro-treated mouse branching organoid assay
Branch
branches
Organoid body
Puromycin staining mean intensity of branches / organoid body
Translation in mouse organoid branches
e
Parental
WT
3SA
Puromycin staining mean intensity of protrusion / cyst
Cyst
Protrusion
f
Bicistronic fluorescent reporter genes
cap-dependent
IRES-dependent
CMV
EYFP
IRES
ECFP
F-actin
EYFP
ECFP
WT
3SA
Protrusion
Cyst
Protrusion
Fluorescence intensity ratio of protrusion / cyst
***

Supplementary Figure 8. Puromycinylation SUnSET assay and bicistronic CAP-dependent and -independent translation assay

a) Tukey boxplots showing quantitation ratio of the intensity in protrusion relative to intensity in cyst from Fig. 6b. **b)** Top: Representative western blot of lysates from MCF10A 3D tubulogenesis assay (day 6) treated in culture with puromycin and then processed for western blotting. Blot was probed with anti-puromycin to identify puromycinylated peptides. No puromycin treatment and cycloheximide (CHX) pre-treatment were used as controls. Bottom: Quantitation of relative puromycin intensity shows similar levels between the groups. Bar plots for the blots from 3 independent experiments (mean ± SD). **c)** Similar to b for mouse 3D branching organoids assay lysates (day 6). Bar plots for the blots from 3 independent experiments (mean ± SD). **d)** Top: Representative immunofluorescence images of mouse 3D organoids (day 3) treated with puromycin in culture, then processed for immunofluorescence and stained with anti-puromycin (red), phalloidin (F-actin, green) and DAPI (blue). Scale bars = 20µm. Bottom: Tukey boxplots for ratio of puromycin intensity in branches relative to intensity in organoid body shows decreased puromycin staining in branches of Bad^{3SA} organoids (blue). $Bad^{+/+}$ *n*=105, Bad^{3SA} *n*=102 from 3 independent experiments. **e)** Tukey boxplots showing quantitation ratio of the puromycin intensity in protrusion relative to intensity in cyst from Fig. 6c. **f)** Top: Schematic of the bicistronic fluorescent reporter genes for cap-dependent translation (EYFP) and cap-independent translation (IRES-dependent ECFP). Bottom: Representative immunofluorescence images from WT (upper) and 3SA (lower) early tubulogenesis assays (day 3) for cells transfected with the bicistronic fluorescent reporter genes, stained with phalloidin (F-actin). The teal line is placed to outline subcellular protrusions subject to subsequent quantification analyses. Right: Tukey boxplots show quantitation for normalized EYFP or ECFP fluorescence intensity ratio in protrusion relative to intensity in cyst. 3SA perturbed both cap-dependent and cap-independent translation, specifically in the protrusions. WT *n*=34, 3SA *n*=37, from 3 independent experiments. For Tukey boxplots, constriction indicates median, notch indicates 95% confidence interval, box edges are 25th and 75th percentiles, whiskers show extreme data points, 'outliers' plotted as black dots, overlaid circle and error bars are the mean ± SEM. For all *p*-values, *** $P<0.001$, ** $P<0.01$, * $P<0.05$. Statistical test details and exact *p*-values are provided in Sup. Data 4. Source data are provided in the Source Data file.

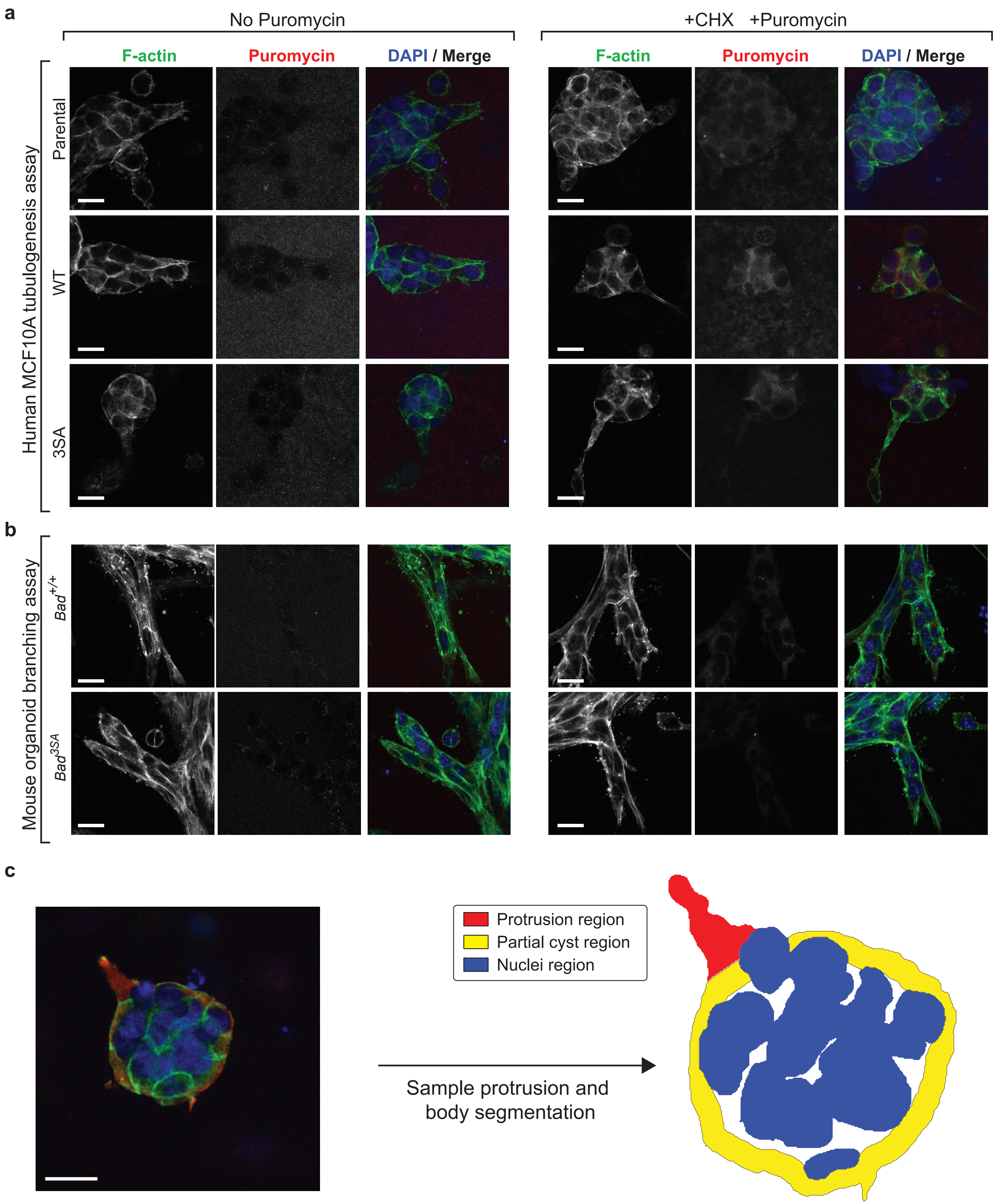
a
No Puromycin
+CHX +Puromycin
F-actin
Puromycin
DAPI / Merge
F-actin
Puromycin
DAPI / Merge
Human MCF10A tubulogenesis assay
Parental
WT
3SA
b
Mouse organoid branching assay
Bad+/+
Bad3SA
c
Protrusion region
Partial cyst region
Nuclei region
Sample protrusion and
body segmentation

Supplementary Figure 9. Puromycin assay controls

a) Representative immunofluorescence images from 3 independent experiments, of tubulogenesis assay controls stained with anti-puromycin (red), phalloidin (F-actin, green) and DAPI (blue) in the absence of puromycin treatment or with cycloheximide (CHX) pre-treatment prior to puromycin treatment. **b)** Representative immunofluorescence images from 3 independent experiments, of puromycin assay controls for mouse 3D organoid branching gels, similar to a. **c)** Representative image segmentation (from Fig.6c, Parental *n*=57, WT *n*=63, 3SA *n*=60 from 3 independent experiments) for protrusion, cyst intensity ratio calculations. Scale bars = 20µm.
