# Supplementary material for: BAD regulates mammary gland morphogenesis by 4E-BP1-mediated control of localized translation in mouse and human models
Source: Nat Commun. 2021 May 19;12:2939. doi: 10.1038/s41467-021-23269-8 (PMC8134504; doi:10.1038/s41467-021-23269-8)
Supplement: Supplementary file 2 — Description of Additional Supplementary Files [file 41467_2021_23269_MOESM2_ESM.pdf]

Title: Supplementary Data 1. Gene Ontology (GO) enrichment analysis from *Bad*$^{+/+}$ and *Bad*^{3SA} pubertal mammary gland MS data

Description: GO enrichment analysis of differentially expressed proteins from the MS screen (*Bad*$^{+/+}$ 5wk vs *Bad*$^{+/+}$ 4wk and *Bad*$^{+/+}$ 5wk vs *Bad*^{3SA} 5wk in different sheets) highlighting significant molecular function (MF) and cellular component (CC) hits (See 'Methods'). Comparative proteomic data analysis is also included in the other sheets.

Title: Supplementary Data 2. Statistical analysis of RPPA data from *Bad*$^{+/+}$ and *Bad*^{3SA} pubertal mammary glands

Description: RPPA screen with target protein/phosphoprotein ranked from most significant analysis of variance *p*-value, comparing *Bad*$^{+/+}$ 4wk, *Bad*$^{+/+}$ 5wk and *Bad*^{3SA} 5wk. Adjusted *p*-value from subsequent Tukey Kramer's post hoc test, $\log_2$ fold change, mean and standard deviation are listed for each protein/phosphoprotein. RPPA validated antibodies have a unique highlight. RPPA raw and normalized data is provided in the subsequent sheets.

Title: Supplementary Data 3. Wiki pathway analysis from *Bad*$^{+/+}$ and *Bad*^{3SA} pubertal mammary gland RPPA data

Description: Top twenty pathways from RPPA pathway analysis (See 'Methods' and Enrichr web application algorithm for details on pathway enrichment, *p*-values and combined score computation[103].). Each pathway (Wiki Pathway ID column) is hyperlinked to its schematic online.

Title: Supplementary Data 4. Antibody resource list and and *p*-values list

Description: List of all antibodies used in this study and *p*-values for all the figures. For the antibodies sheet, the catalog numbers are hyperlinked to the source company respective antibody webpage. Link to all RPPA antibodies used is included. For the *p*-values sheets, significant *p*-values are highlighted in yellow.

Title: Supplementary Movie 1. 3SA has unstable subcellular protrusions

Description: MCF10A 3D tubulogenesis time lapse assay is shown. Nascent protrusions were imaged for 24 hours, starting at day 3. Stable and unstable protrusions are marked with red and green asterisks, respectively.

Title: Supplementary Movie 2. Protrusion instability in 3SA is not caspase dependent

Description: 3D tubulogenesis assays were control-treated, or treated with z-VAD-fmk or ABT-737 as indicated and imaged, starting at day 3. Time lapse images were collected for 24 hours. Stable and unstable protrusions are marked with red and green asterisks, respectively.

Title: Supplementary Movie 3. 4E-BP1 knockdown stabilizes protrusions

Description: MCF10A 3D tubulogenesis time lapse assay is shown. Nascent protrusions were imaged for 24 hours, starting at day 3. Stable and unstable protrusions are marked with red and green asterisk, respectively.
