# Supplementary material for: BAD regulates mammary gland morphogenesis by 4E-BP1-mediated control of localized translation in mouse and human models
Source: Nat Commun. 2021 May 19;12:2939. doi: 10.1038/s41467-021-23269-8 (PMC8134504; doi:10.1038/s41467-021-23269-8)
Supplement: Supplementary file 11 — Reporting Summary [file 41467_2021_23269_MOESM11_ESM.pdf]

nature research

Corresponding author(s): Ing Swie Goping

Last updated by author(s): Mar 23, 2021

Reporting Summary

Nature Research wishes to improve the reproducibility of the work that we publish. This form provides structure for consistency and transparency in reporting. For further information on Nature Research policies, see our Editorial Policies and the Editorial Policy Checklist.

Statistics

For all statistical analyses, confirm that the following items are present in the figure legend, table legend, main text, or Methods section.

n/a	Confirmed	
☐	☒	The exact sample size (n) for each experimental group/condition, given as a discrete number and unit of measurement
☐	☒	A statement on whether measurements were taken from distinct samples or whether the same sample was measured repeatedly
☐	☒	The statistical test(s) used AND whether they are one- or two-sided<br>*Only common tests should be described solely by name; describe more complex techniques in the Methods section.*
☒	☐	A description of all covariates tested
☐	☒	A description of any assumptions or corrections, such as tests of normality and adjustment for multiple comparisons
☐	☒	A full description of the statistical parameters including central tendency (e.g. means) or other basic estimates (e.g. regression coefficient) AND variation (e.g. standard deviation) or associated estimates of uncertainty (e.g. confidence intervals)
☐	☒	For null hypothesis testing, the test statistic (e.g. F, t, r) with confidence intervals, effect sizes, degrees of freedom and P value noted<br>*Give P values as exact values whenever suitable.*
☒	☐	For Bayesian analysis, information on the choice of priors and Markov chain Monte Carlo settings
☒	☐	For hierarchical and complex designs, identification of the appropriate level for tests and full reporting of outcomes
☒	☐	Estimates of effect sizes (e.g. Cohen's d, Pearson's r), indicating how they were calculated

Our web collection on statistics for biologists contains articles on many of the points above.

Software and code

Policy information about availability of computer code

Data collection	Proteome Discoverer™ 1.4.1.14<br>Epson Perfection scanner v1650 GUI<br>BD Accuri™ C6<br>Microsoft Office16 Word<br>Microsoft Office16 Excel<br>GraphPad Prism version 9.0.0 (86)<br>Image Studio™ version 5.2
Data analysis	MATLAB R2018a<br>GraphPad Prism version 9.0.0 (86)<br>Proteome Discoverer™ 1.4.1.14's SEQUEST search engine<br>Enrichr web application<br>FlowJo (v10)

For manuscripts utilizing custom algorithms or software that are central to the research but not yet described in published literature, software must be made available to editors and reviewers. We strongly encourage code deposition in a community repository (e.g. GitHub). See the Nature Research guidelines for submitting code & software for further information.

Data

Policy information about availability of data

All manuscripts must include a data availability statement. This statement should provide the following information, where applicable:

- Accession codes, unique identifiers, or web links for publicly available datasets
- A list of figures that have associated raw data
- A description of any restrictions on data availability

The mass spectrometry proteomics data have been deposited to the ProteomeXchange Consortium via the PRIDE (PubMed ID: 30395289) partner repository with the dataset identifier PXD014347 (https://www.ebi.ac.uk/pride/archive/projects/PXD014347). The comparative proteomic data analysis has been included in Sup. Data 1. (Mus musculus proteome retrieved from UniprotKB 'release-2015_10' version on October 16, 2015).

Field-specific reporting

Please select the one below that is the best fit for your research. If you are not sure, read the appropriate sections before making your selection.

- [x] Life sciences
- [ ] Behavioural & social sciences
- [ ] Ecological, evolutionary & environmental sciences

For a reference copy of the document with all sections, see nature.com/documents/nr-reporting-summary-flat.pdf

Life sciences study design

All studies must disclose on these points even when the disclosure is negative.

Sample size	No formal sample size calculation was performed. In experiments involving cell imaging analyses where biological material was not limiting, sample size was n>10. With significance set at alpha=0.05 and power of 0.8, this would identify a difference of 50% between groups, which is an acceptable range of scientific interest. In analyses using animals, reduction of numbers was a priority such that n >5 independent animals was chosen. We accepted a larger effect difference would be identified.
Data exclusions	No data was excluded from the analyses.
Replication	All reasonable measures were taken to verify reproducibility of experimental findings. Such measures included use of tissues or cells that were independently harvested for biological replicates. All attempts at replication were successful. A minimum of 3 independent experiments were performed and included in all the figures.
Randomization	Randomization is not relevant. Samples were allocated into experimental groups based on genotype.
Blinding	Since data collection and analyses are quantitative and not qualitative, blinding is not relevant to this study.

Reporting for specific materials, systems and methods

We require information from authors about some types of materials, experimental systems and methods used in many studies. Here, indicate whether each material, system or method listed is relevant to your study. If you are not sure if a list item applies to your research, read the appropriate section before selecting a response.

Materials & experimental systems

n/a	Involved in the study	
[ ]	[x]	Antibodies
[ ]	[x]	Eukaryotic cell lines
[x]	[ ]	Palaeontology and archaeology
[ ]	[x]	Animals and other organisms
[x]	[ ]	Human research participants
[x]	[ ]	Clinical data
[x]	[ ]	Dual use research of concern

Methods

n/a	Involved in the study	
[x]	[ ]	ChIP-seq
[ ]	[x]	Flow cytometry
[x]	[ ]	MRI-based neuroimaging

Antibodies

Antibodies used	All antibodies (primary and secondary) and fluorescent markers are listed below. Supplementary Data 4 contains this information together with their source, validation, dilution as well as hyperlink to their supplier web page. For the 246 antibodies probed by MD Anderson RPPA services, refer to Supplementary Data 2 and Supplementary Data 4.<br><br>Primary antibodies<br>Mouse-anti-CK14 ; abcam --Cat# ab7800

Mouse-anti-Paxillin ; BD biosciences --Cat# 610051
Mouse-anti-pS112-BAD ; CST --Cat# 9296
Mouse-anti-Puromycin ; Sigma --Cat# MABE343
Mouse-anti-Tubulin ; Sigma --Cat# T5168
Rabbit-anti-4E-BP1 ; CST --Cat# 9644
Rabbit-anti-Akt ; CST --Cat# 9272
Rabbit-anti-BAD ; Sigma --Cat# B0684
Rabbit-anti-BclXL ; CST --Cat# 2762
Rabbit-anti-Calnexin ; Enzo --Cat# ADI-SPA-860
Rabbit-anti-Caspase 3 ; Enzo --Cat# ADI-AAP-113
Rabbit-anti-CD24-PE ; StemCell Technologies --Cat# 60099PE, 60099PE.1
Rabbit-anti-CD49f-FITC ; StemCell Technologies --Cat# 60037FI, 60037FI.1
Rabbit-anti-CK18 ; Thermo Fisher Scientific --Cat# MA5-15458
Rabbit-anti-cleaved Caspase 3 ; CST --Cat# 9664
Rabbit-anti-eIF4E ; CST --Cat# 9742
Rabbit-anti-eIF4G ; CST --Cat# 2498
Rabbit-anti-mouse CD326 (Ep-CAM) -647 ; BioLegend --Cat# 118212
Rabbit-anti-ERK1/2 ; Santa Cruz --Cat# sc-93
Rabbit-anti-mTOR ; CST --Cat# 2983
Rabbit-anti-p70S6K ; CST --Cat# 2708
Rabbit-anti-pS112-BAD ; CST --Cat# 9291
Rabbit-anti-pS136-BAD ; abcam --Cat# ab28824
Rabbit-anti-pS136-BAD ; CST --Cat# 5286
Rabbit-anti-pS2448-mTOR ; CST --Cat# 2971
Rabbit-anti-pS473-Akt ; CST --Cat# 4058
Rabbit-anti-pS65-4E-BP1 ; CST --Cat# 9456
Rabbit-anti-pT389-p70S6K ; CST --Cat# 9205
Rabbit-anti-T202/Y204-ERK1/2 ; CST --Cat# 9101
Rabbit-anti-T37/46-4E-BP1 ; CST --Cat# 2855
Rabbit-anti-Tom20 ; Santa Cruz --Cat# sc-11415

Secondary antibodies
Donkey-anti-mouse Alexa Fluor® 488 ; Thermo Fisher Scientific --Cat# A21202
Donkey-anti-mouse Alexa Fluor® 555 ; Thermo Fisher Scientific --Cat# A31570
Donkey-anti-mouse Alexa Fluor® 647 ; Thermo Fisher Scientific --Cat# A31571
Donkey-anti-rabbit Alexa Fluor® 647 ; Thermo Fisher Scientific --Cat# A31573
Goat-anti-mouse Alexa Fluor® 680 ; Thermo Fisher Scientific --Cat# A21057
Goat-anti-rabbit Alexa Fluor® 488 ; Thermo Fisher Scientific --Cat# A11008
Goat-anti-rabbit Cy3 ; abcam --Cat# ab97075

Fluorescent markers
DAPI (4',6-Diamidino-2-Phenylindole, Dihydrochloride. Nucleus marker) ; Thermo Fisher Scientific --Cat# D1306
Phalloidin-Alexa Fluor® 647 (F-actin marker) ; Thermo Fisher Scientific --Cat# A22287

Validation

Validation details are updated in supplementary Data 2 and Supplementary Data 4

Eukaryotic cell lines

Policy information about cell lines

Cell line source(s)	MCF10A parental cell line was purchased from ATCC (invoice provided during review process)
Authentication	As per ATCC standard practice, our purchased MCF10A cell line was authenticated using Short Tandem Repeat (STR) Profiling, Cellular Morphology, Karyotyping and Cytochrome C Oxidase I (COI) Assay Testing.
Mycoplasma contamination	We routinely test with a mycoplasma-specific PCR test (Eldering et al. 2004, Biologicals: Journal of the International Association of Biological Standardization 32: 183) and demonstrate that the cells are free of mycoplasma.
Commonly misidentified lines (See ICLAC register)	None

Animals and other organisms

Policy information about studies involving animals; ARRIVE guidelines recommended for reporting animal research

Laboratory animals

All mouse strains were in the C57BL/6J background. Only female mice were used, their age is listed below:
Fig 1a-b, Puberty 5wk; Adult 12wk; Pregnant and Involution 8-12wk.
Fig 2a-c, Pubertal onset 4wk; Puberty 5wk; End of puberty 8wk; Old virgin 40-43wk. Fig 2e, Pre-puberty 3wk; Donor tissue 8wk.

	Fig 3b-d, Primary Organoids source, pubertal 5wk mice.<br>Fig 5a-d, Pubertal onset 4wk; Puberty 5wk. Fig 5e, Primary Organoids source, pubertal 5wk mice.<br>Sup Fig 1a-f, Pubertal onset 4wk; Puberty 5wk; End of puberty 8wk.<br>Sup Fig 2a-f, Pre-puberty 3wk; Puberty 5wk; End of puberty 8wk; Old virgin 40-43wk; Pregnant and Involution 8-12wk.<br>Sup Fig 3a-c, Primary Organoids source, pubertal 5wk mice.<br>Sup Fig 4a, Pubertal onset 4wk; Puberty 5wk.<br>Sup Fig 5a, Puberty 5wk.<br>Sup Fig 6a-c, Primary epithelial cells source for flow cytometry, 8wk mice.<br>Sup Fig 7a-f, Pubertal onset 4wk; Puberty 5wk; Primary Organoids source, pubertal 5wk mice.<br>Sup Fig 8c-d, Primary Organoids source, pubertal 5wk mice.<br>Sup Fig 9b, Primary Organoids source, pubertal 5wk mice.
Wild animals	The study did not involve wild animals
Field-collected samples	The study did not involve samples collected from the field
Ethics oversight	Animal procedures were performed in accordance with the guidelines and regulations set forth by the Canadian Council on Animal Care and approved by the University of Alberta Health Sciences 2 Animal Care and Use Committee (Protocol# AUP00000386). Mice were kept in a temperature (22C) and humidity (40-60%) controlled room on a 14-hour light/10-hour dark cycle.

Note that full information on the approval of the study protocol must also be provided in the manuscript.

Flow Cytometry

Plots

Confirm that:

- [x] The axis labels state the marker and fluorochrome used (e.g. CD4-FITC).
- [x] The axis scales are clearly visible. Include numbers along axes only for bottom left plot of group (a 'group' is an analysis of identical markers).
- [x] All plots are contour plots with outliers or pseudocolor plots.
- [x] A numerical value for number of cells or percentage (with statistics) is provided.

Methodology

Sample preparation	8-wk mouse mammary glands were minced using a sterile blade and subsequently digested in dissociation medium (20mg Collagenase A and 10mg Dispase II in 10ml DMEM/F12) for 3 hours at 37oC with 120 rpm shaking. The dissociated tissue was spun down at 450g for 10 minutes and then supernatant was discarded. Preparation of a single cell suspension was done as detailed (Prater, M., Shehata, M., Watson, C. J. & Stingl, J. Enzymatic dissociation, flow cytometric analysis, and culture of normal mouse mammary tissue. Methods Mol. Biol. 946, 395-409 (2013)).
Instrument	BD Accuri™ C6 flow cytometer
Software	BD Accuri™ C6 flow cytometer software was used to collect data, FlowJo (v10) was used to analyze the data
Cell population abundance	EasySep™ Mouse Epithelial Cell Enrichment Kit II (Stem Cell Technologies) was used to exclude non-epithelial (lineage negative) cells following the manufacturer's instructions. This kit specifically uses immunomagnetic negative selection. The EasySep™ procedure involves labeling unwanted non-epithelial cells with biotinylated antibodies and magnetic particles. The magnetically labeled cells are separated from untouched desired cells by using an EasySep™ magnet and simply pouring the desired cells into a new tube.
Gating strategy	FSC-A/SSC-A gate (see Supp. Fig 6a) was used to gate for cells. FSC-A/FSC-H gate (see Supp. Fig 6a) was used to gate for single cells. Luminal cell populations were gated with EpCAMhighCD49fmed or CD24highCD49fmed while basal cell populations were gated with EpCAMmedCD49fhigh or CD24medCD49fhigh (see Supp. Fig 6b-c). These markers were used because they had been shown to have the least tendency of contaminating gated basal cells with non-epithelial cells, especially in the C57BL/6J background, which is the strain of mice used in our study (Gao, H. et al. Murine mammary stem/progenitor cell isolation: Different method matters? Springerplus 5, 140-3. eCollection 2016 (2016)).

- [x] Tick this box to confirm that a figure exemplifying the gating strategy is provided in the Supplementary Information.
